# Machine learning prediction of the mechanical properties of injection-molded polypropylene through X-ray diffraction analysis

**DOI:** 10.1080/14686996.2024.2388016

**Published:** 2024-08-05

**Authors:** Ryo Tamura, Kenji Nagata, Keitaro Sodeyama, Kensaku Nakamura, Toshiki Tokuhira, Satoshi Shibata, Kazuki Hammura, Hiroki Sugisawa, Masaya Kawamura, Teruki Tsurimoto, Masanobu Naito, Masahiko Demura, Takashi Nakanishi

**Affiliations:** a Materials Open Platform for Chemistry, National Institute for Materials Science, Ibaraki, Japan; b Center for Basic Research on Materials, National Institute for Materials Science, Ibaraki, Japan; c Process Technology Laboratory, R&D Center, Mitsui Chemicals, Inc., Chiba, Japan; d Essential Chemicals Research Laboratory, Sumitomo Chemical Co., Chiba, Japan; e Platform Laboratory for Science & Technology, Asahi KASEI Corporation, Shizuoka, Japan; f Science & Innovation Center, Mitsubishi Chemical Corporation, Kanagawa, Japan; g Research Center for Macromolecules and Biomaterials, National Institute for Materials Science, Ibaraki, Japan; h Research Network and Facility Services Division, National Institute for Materials Science, Ibaraki, Japan; i Research Center for Materials Nanoarchitectonics (MANA), National Institute for Materials Science, Ibaraki, Japan

**Keywords:** Polypropylene, X-ray diffraction, bayesian spectral deconvolution, ising machine, machine learning

## Abstract

Predicting the mechanical properties of polymer materials using machine learning is essential for the design of next-generation of polymers. However, the strong relationship between the higher-order structure of polymers and their mechanical properties hinders the mechanical property predictions based on their primary structures. To incorporate information on higher-order structures into the prediction model, X-ray diffraction (XRD) can be used. This study proposes a strategy to generate appropriate descriptors from the XRD analysis of the injection-molded polypropylene samples, which were prepared under almost the same injection molding conditions. To this end, first, Bayesian spectral deconvolution is used to automatically create high-dimensional descriptors. Second, informative descriptors are selected to achieve highly accurate predictions by implementing the black-box optimization method using Ising machine. This approach was applied to custom-built polymer datasets containing data on homo- polypropylene and derived composite polymers with the addition of elastomers. Results show that reasonable accuracy of predictions for seven mechanical properties can be achieved using only XRD.

## IMPACT STATEMENT

This study proposes a strategy to generate appropriate descriptors, which realize highly accurate predictions of mechanical properties via machine learning from the XRD analysis of the molded polypropylene samples.

## GRAPHICAL ABSTRACT

**Figure:**
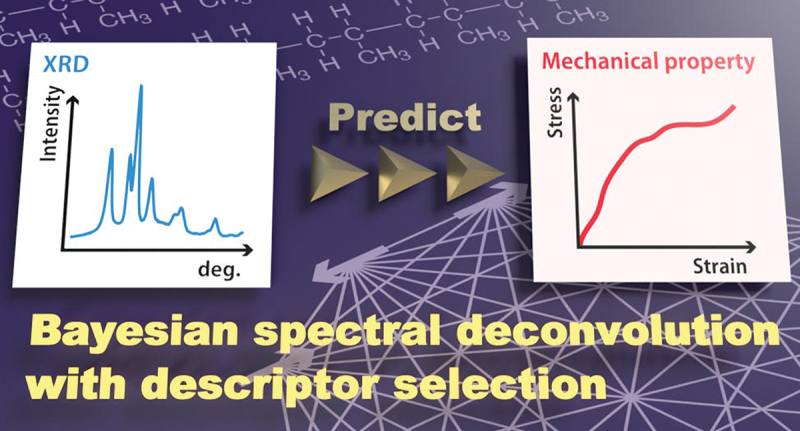

## 1. Introduction

Machine learning (ML) is powerful and indispensable for materials design [1–8]. ML can be applied to data on existing materials to predict the properties of unknown materials. However, to achieve accurate predictions, it is essential to use descriptors that correctly represent the features of these materials. Numerous ML tools have been developed to create descriptors for organic [9–11], polymer [12–14], and inorganic materials [15–17]. These descriptors are generally based on variables that researchers can easily control and generate without the need for actual synthesis, such as element concentration, materials structure, and manufacturing processes.

However, predicting the mechanical properties of polymer materials, particularly those of crystalline polymers containing higher-order structures, is challenging even with the power of ML. This is because not only the structure and degree of monomer polymerization that can be controlled but also the higher-order structure and morphology after molding significantly affect the mechanical properties of the material [18,19]. Therefore, predicting the mechanical properties of a polymer material after molding from just the monomer information and manufacturing process is typically challenging [20]. This encumbers the design of desired polymer materials based on ML. However, it is possible to predict the mechanical properties using descriptors that reflect the higher-order structural information of the polymer after molding. For example, higher-order structures can be captured by X‐ray diffraction (XRD) measurements [21], and if the XRD results can be used as descriptors, the prediction of mechanical properties will be credible. However, XRD results can only be obtained after molding and the mechanical properties of unknown polymer materials cannot be predicted using these results as descriptors. An advantage of predicting mechanical properties from XRD results is the possibility of substituting complex destructive test measurements with simple non-destructive counterparts. In particular, by combining this predictor with the latest high-throughput polymer synthesis technology [22], polymer materials with desired mechanical properties can be screened using only XRD measurements, which contributes to a faster polymer design.

When using the XRD results as descriptors, the part of the diffraction pattern that captures the structural information should be selected appropriately. Certainly, using the entire diffraction pattern is possible, but typically, an excessively large descriptor dimensionality may lead to overfitting and reduce the prediction accuracy. Therefore, to achieve highly accurate predictions using XRD analysis, the extraction of parameters that express the shape of the diffraction pattern is crucial. For the important parameters, the shape of each peak would be a better descriptor. However, extracting the number of peaks from the XRD results for polymer materials is challenging. To automatically extract this number, Bayesian spectral deconvolution has been proposed as a general method for analyzing spectra in materials science [23–25]. Using the exchange Monte Carlo approach [26], the number of peaks and their shapes are automatically obtained.

**Figure 1.**
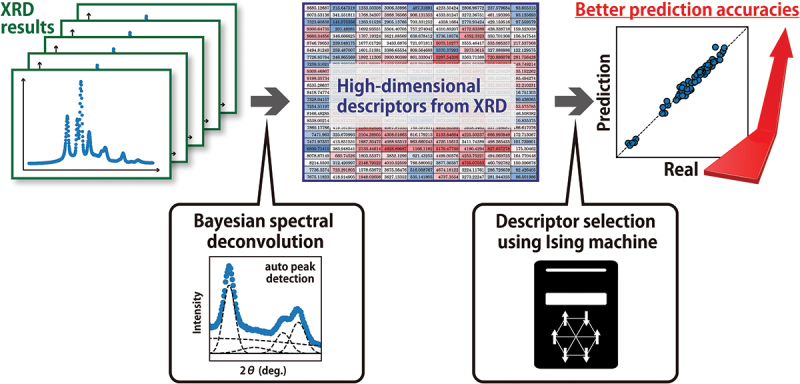
Flow diagram of the procedure used to provide a highly accurate machine learning prediction model using only the XRD results of polymer materials.

In this study, we prepared two types of datasets of injection-molded polypropylene samples, polypropylene (PP)s and composites of PP incorporating elastomers, under almost the same injection molding conditions. For injection molding, the same condition was used for composites of PP incorporating elastomers, while the temperature was changed only for PPs. Since the machine learning approach for polymer materials is difficult due to the hierarchical structure, the influence of the molding process on the higher-order structures was avoided as much as possible in our dataset. Bayesian spectral deconvolution was applied for obtained XRD patterns. By performing the machine learning analysis separately for two datasets, the extracted descriptors were used as ML models to predict the mechanical properties. It is shown that highly accurate predictions can be achieved by appropriately selecting the descriptors obtained through Bayesian spectral deconvolution. In addition, a black-box optimization method based on an Ising machine [27,28] was used to select the informative descriptors. With this procedure, most of the nine different mechanical properties could be predicted from the XRD results alone, excluding elongation at break and Rockwell hardness. The results indicate that machine learning can be effectively applied to the polymer materials dataset by preparing the dataset with the same conditions as possible. Thus, we believe this study becomes a test case for future data-driven approaches to polymer science. The procedure used in this study is illustrated in Figure 1. Note that, although descriptor selection is performed, the analysis of the relationship between the selected descriptors and the mechanical properties is not the purpose of this study.

## 2. Polypropylene dataset

Two types of custom-built polymer datasets were used to test this approach. The first dataset contained 15 types of homo-polypropylene (homo-PP) materials and is referred to as the homo-PP dataset. For each material, five temperatures (30, 45, 60, 70, and 75°C) were used for injection molding, and a total of 75 data were generated, which are summarized in Table S1. We confirmed that the XRD results do not change significantly when the temperature is changed, and the XRD results of an example are shown in Figure S1. Thus, we considered that the samples with different temperatures can be treated as samples obtained under the same injection molding conditions. For the second dataset, referred to as the homo-PP-with-elastomer dataset, four types of homo-PPs with five different elastomers were targeted. The injection molding temperature was fixed at 45°C. Polymer materials were prepared with different mixing ratios (homo-PP:elastomer = 9:1, 8:1, and 7:1), using three types of screws (S1: weak, S2: medium, and S3: strong) for the twin screw extruder (Berstorff, ZE40A, Germany) and two different rotational speeds (100 and 300 rpm). A total of 120 samples were prepared, the details of which are summarized in Table S2. For each polymer material, a dumbbell-shaped specimen with a thickness of 4 mm was prepared by injection molding, and nine types of mechanical properties were measured for each sample: tensile modulus (MPa), yield stress (MPa), yield strain (%), elongation at break (%), flexural strength (MPa), flexural modulus (MPa), Charpy impact value (kJ/m^2^), heat distortion temperature (°C), and Rockwell hardness (HRC). Tensile tests (5966 Universal Testing Systems, Instron®, US) were performed at a tensile speed of 50 mm/min and a chuck distance of 115 ± 1 mm. In addition, the Charpy impact test (Digital Impact Testing Machine DG-UB Type, Toyo Seiki Seisaku-sho, Ltd., Japan) was performed at 23°C, the heat distortion temperature (HDT Tester 3M-2V, Toyo Seiki Seisaku-sho, Ltd., Japan) was measured at 0.45 MPa, and the Rockwell hardness (Rockwell Hardness Tester Model 3R, Imai Testing Machine MFG., Co., Ltd., Japan) was measured while overlapping two samples. Each test was repeated five times and the mean values of the results were used as the prediction target. XRD measurements in the machine direction (MD; flow direction of the resin) and transverse direction (TD; width direction of the resin) were performed using a Rigaku SmartLab (Rigaku, Japan), which has a PhotonMax high-flux 9 kW rotating-anode X-ray source coupled with a HyPix-3000 high-energy-resolution semiconductor detector.

## 3. Results

### 3.1. Bayesian spectral deconvolution of XRD results

**Figure 2.**
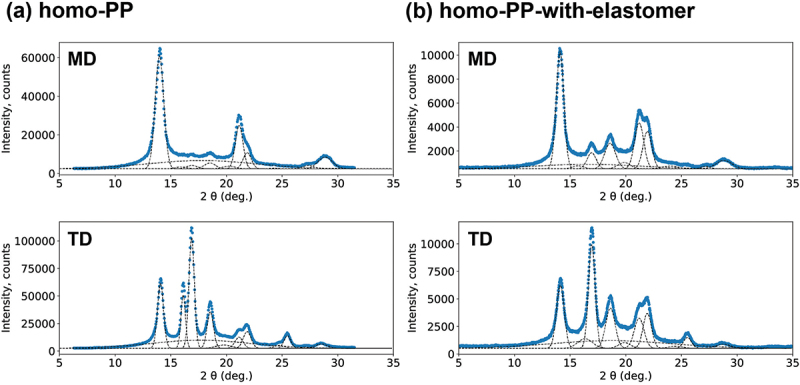
Examples of the Bayesian spectral deconvolution results for (a) homo-pp and (b) homo-pp with an elastomer when the XRD results of MD and TD, respectively, are targeted. The target samples are the ID 1 for each dataset. The blue points are the measured data, and the dotted lines are the fitting results.

Bayesian spectral deconvolution was used to extract descriptors from the XRD results. Initially, the target peak function was defined as a Gaussian function, and the number of peaks was established to minimize the Bayesian free energy calculated using the exchange Monte Carlo method. Consequently, for each direction, that is, MD and TD, 12 and 11 peaks, including an amorphous halo, were extracted from the XRD data for the homo-PP and the homo-PP-with-elastomer datasets, respectively. Subsequently, a Gaussian function is estimated for each peak. Five parameters characterizing each peak – that is, the height, location, full width at half maximum (FWHM), area, and area ratio of the peaks – were extracted. Thus, for the homo-PP dataset, 120-dimensional descriptors were obtained, including MD and TD, and the dimensions of the descriptors for the homo-PP with the elastomer dataset were 110. Examples of the Bayesian spectral deconvolution results for homo-PP and homo-PP with an elastomer are shown in Figure 2. Although the five parameters in each Gaussian peak are not independent, we do not know in advance which are better parameters to improve the prediction accuracy of mechanical properties. Thus, after preparing such redundant features, the strategy of selecting important features, which will be explained in Sec. 3.2, was adopted in this study.

### 3.2. Descriptor selection

The Bayesian spectral deconvolution technique allowed the extraction of descriptors with more than 100-dimensions from the XRD results. However, this dimensionality is too high, resulting in overfitting and poor prediction accuracy at this rate. The prediction performances using all the extracted descriptors are shown in Table S3, where the failure in the predictions of almost all the mechanical properties is revealed. Therefore, the appropriate selection of the descriptors that can be used is essential to improve the prediction accuracy. The least absolute shrinkage and selection operator (LASSO) method is a well-known feature selection method used to avoid overfitting in linear regression; however, LASSO is often trapped in local minimum [29], and is seldom a suitable method. The exhaustive-search (ES) method [30,31] is useful for finding better combinations of descriptors as it exhaustively searches for the optimal combination that achieves the highest prediction accuracy. However, in the case of the homo-PP, $2^{120}-1$ combinations existed when considering all the combinations of descriptors, and the ES method could not be used. Therefore, this study used a different approach to searching for better combinations. Finding a better combination to improve the prediction accuracy can be considered as a binary black-box optimization. A factorization machine with quantum annealing (FMQA) algorithm [27] using Ising machines was proposed as a binary black-box optimization method that can be used to select appropriate XRD descriptors. The procedure for selecting descriptors using FMQA is as follows:

1. The dimension of the descriptors is N. Ten types of binary bits of length N are randomly generated. If the binary bit for each bit string is 1, the corresponding descriptor is used, and the mean squared error (MSE), evaluated by 10-fold cross-validation, is calculated. Linear regression is used as the ML prediction model. These 10 types of data are the initial data for black-box optimization.
2. The factorization machine (FM) is trained with 10 data points to predict the MSE when a binary string is input. The FM is defined as follows:
  (1) $$\mathrm{MSE}=\overset{N}{\underset{i=1}{\sum }}w_{i}^{*}q_{i}+\overset{N}{\underset{i=1}{\sum }}\overset{N}{\underset{j=1}{\sum }}\overset{K}{\underset{k=1}{\sum }}v_{ik}^{*}v_{jk}^{*}q_{i}q_{j},$$
  where $q_{i}$ is the 0/1 binary bit, and the value of K is fixed at 8. The trained parameters are written as $w_{i}^{*}$ and $v_{ik}^{*}$
3. By solving this FM with an Ising machine, (in this study, Fixstars Amplify AE[32] is used), a set of descriptors that would reduce the MSE is searched.
4. For the selected descriptors, we evaluate the actual value of the MSE by training a linear regression model.
5. Steps 2-4 are repeated for 290 times, and this independent trial is performed five times.

Since black-box optimization can perform a global search, better combinations can be found compared to LASSO by avoiding entrapment in local minima.

**Figure 3.**
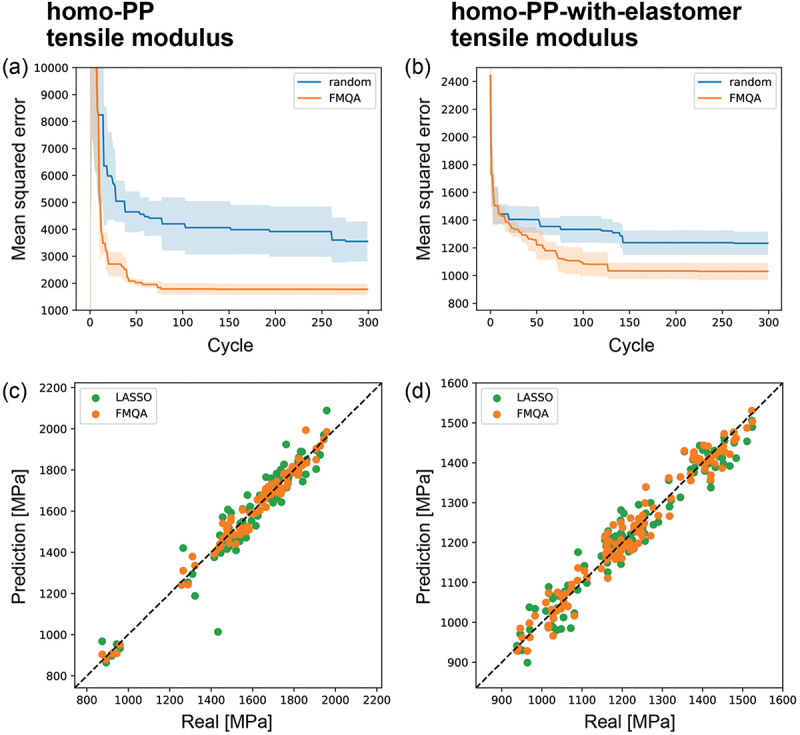
Mean squared error (MSE) as a function of the number of cycles for the tensile modulus of (a) homo-pp and (b) homo-pp-with-elastomer datasets. As the 10-fold cross-validation evaluates the MSE, this value indicates the prediction accuracy. The lines and shaded areas are the mean and standard deviation of five independent trials, respectively. Prediction results using 10-fold cross-validation for the (c) homo-pp and (d) homo-pp-with-elastomer datasets. The lowest MSE case in the FMQA search and LASSO results are shown for comparison.

The results of the descriptor selection for the tensile modulus case are shown in Figure 3(a,b). The horizontal axis represents the number of cycles and the vertical axis represents the corresponding minimum MSE value. The mean MSE values of five independent trials are plotted. For comparison, the results for a randomly generated bit string are also presented. Compared with random sampling, the MSE value of FMQA decreased with each cycle. Thus, FMQA determines a set of descriptors to achieve better prediction accuracy. Figures 3(c,d) present the prediction results of the 10-fold cross-validation of the lowest MSE in the FMQA search. These data points were not included in the training data when predicting each point. Most data points were distributed around the 45-degree line, and better predictions were achieved. The results of LASSO are also plotted for comparison, revealing that FMQA allowed the selection of descriptors to build an ML model with better prediction accuracy. Here, sklearn.linear_model.LassoCV with default settings was used to perform LASSO [33]. We confirmed that the FMQA obtained better descriptors for the other eight mechanical properties (see Figures S2 and S3). As a reference, the selected descriptors depending on the mechanical properties using FMQA and LASSO are summarized in Figures S4–S7. The extraction of additional information regarding the relationship between the descriptors and predicted targets using these results is cumbersome.

### 3.3. Prediction accuracies of mechanical properties by XRD results

**Figure 4.**
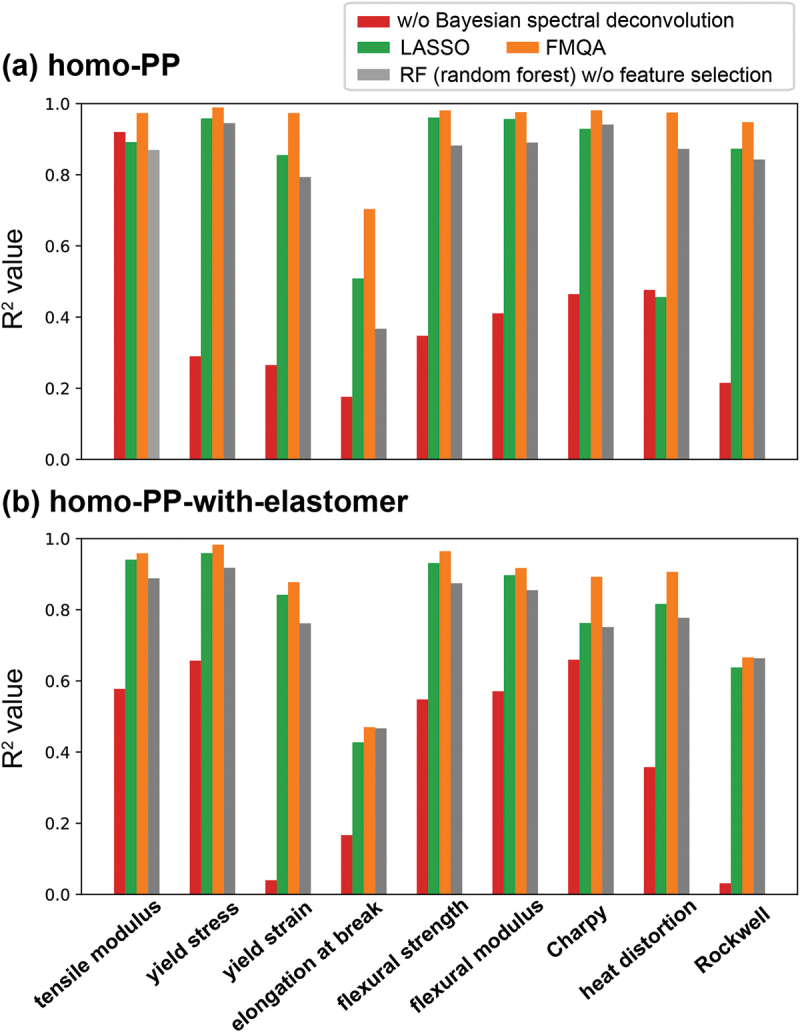
R^2^ values for the predictions when the 10-fold cross-validation is performed for the lowest MSE case in the FMQA search for (a) homo-pp and (b) homo-pp-with-elastomer datasets. We also plot the results obtained using LASSO, the six-dimensional descriptors without using Bayesian spectral deconvolution, and the random forest regression without feature selection. As the R^2^ values approach 1, the prediction accuracy increases.

To illustrate the prediction accuracies for the nine mechanical properties, Figure 4 plots the R^2^ values for the predictions when a 10-fold cross-validation is performed for the lowest MSE case in the FMQA search. The LASSO results are also shown. For comparison purposes, the α(110) peak height, FWHM, and crystallinity, which are usually extracted by researchers without using Bayesian spectral deconvolution, were derived from the XRD results for MD and TD, and the prediction results using these six-dimensional descriptors were obtained. In addition, for comparison with the nonlinear model, the results of the random forest model without the feature selection are also evaluated. Clearly, our Bayesian spectral deconvolution technique increased the prediction accuracy for all the mechanical properties. The selection of descriptors using FMQA ensures that most mechanical properties can be predicted with high accuracy based solely on XRD results (see Table S3). Furthermore, the prediction accuracy of the linear model and the feature selection by FMQA outperform those of the nonlinear random forest model. However, the prediction accuracy for the elongation at break was low for both homo-PP and homo-PP-with-elastomer datasets. We confirmed that the experimental accuracy for the elongation was poor, and that the values changed significantly over the five experiments. For example, in the homo-PP sample with the highest variability, the elongation values over the five experiments were 87.9%, 108.2%, 476.7%, 483.5%, and 491.3%. Thus, the prediction targets have a high uncertainty, rendering accurate predictions impossible. In addition, the Rockwell hardness for homo-PP-with-elastomer dataset has a lower prediction accuracy than the others, although it was not as inaccurate as the elongation. Similar to elongation, this is a property with high variation between samples. These large variations between samples for elongation and Rockwell hardness are due to the structural defects in the samples. However, the XRD data has information on crystal structure and little information on structural defects. Thus, we believe it is difficult to predict the elongation and Rockwell hardness from the XRD results. It should be emphasized that the prediction of the other seven mechanical properties, excluding elongation at break and Rockwell hardness, was achieved with high accuracy based solely on the results of XRD, which is a relatively simple measurement.

## 4. Conclusions

This study attempted to predict the mechanical properties of PPs using XRD analysis alone. To achieve highly accurate predictions, a strategy was proposed using Bayesian spectral deconvolution and an informative descriptor selection method by employing FMQA. Consequently, most of the mechanical properties considered in this study, such as the tensile modulus, yield stress, yield strain, flexural strength, flexural modulus, Charpy impact value, and heat distortion temperature, were predicted with high accuracy using XRD results alone. However, it was challenging to predict the elongation at break. Therefore, our approach enables the prediction of the mechanical properties of PPs, typically assessed through destructive testing, using only nondestructive testing, specifically, XRD. In this study, each dataset was prepared under almost the same injection molding conditions, and the machine learning analysis was performed separately for each dataset. Thus, our trained model cannot be applied to predict the mechanical properties of all PPs. If new PP samples are considered, we need to collect the mechanical properties and prepare the machine learning model according to our proposed procedure.

The combination of Bayesian spectral deconvolution and the informative descriptor selection method using FMQA can be applied to other spectral analyses such as X-ray photoelectron spectroscopy and Mössbauer spectroscopy measurements. Therefore, our approach contributes to the highly accurate prediction of properties that cannot be expressed without incorporating complex information from measurements.

## Supplementary Material

Supplemental Material

## Data Availability

Due to the nature of the research, due to commercial supporting data is not available.

## Impact statement

This study proposes a strategy to generate appropriate descriptors, which realize highly accurate predictions of mechanical properties via machine learning from the XRD analysis of the molded polypropylene samples.
