# Supplementary material for: Machine learning prediction of the mechanical properties of injection-molded polypropylene through X-ray diffraction analysis
Source: Sci Technol Adv Mater. 2024 Aug 5;25(1):2388016. doi: 10.1080/14686996.2024.2388016 (PMC11328794; doi:10.1080/14686996.2024.2388016)
Supplement: Supplemental Material [file TSTA_A_2388016_SM8500.pdf]

Supporting information for

Machine learning prediction of the mechanical properties of injection-molded polypropylene through X-ray diffraction analysis

Ryo Tamura[1,2], Kenji Nagata[1,2], Keitaro Sodeyama[1,2], Kensaku Nakamura[1,3], Toshiki Tokuhira[1,3], Satoshi Shibata[1,4], Kazuki Hammura[1,5], Hiroki Sugisawa[1,6], Masaya Kawamura[1,6], Teruki Tsurimoto[1,6], Masanobu Naito[1,7], Masahiko Demura[1,8,2], and Takashi Nakanishi[1,9]

[1]Materials Open Platform for Chemistry, National Institute for Materials Science, Ibaraki 305-0044, Japan

[2]Center for Basic Research on Materials, National Institute for Materials Science, Ibaraki 305-0044, Japan

[3]Process Technology Laboratory, R&D Center, Mitsui Chemicals, Inc., Chiba 299-0265, Japan

[4]Essential Chemicals Research Laboratory, Sumitomo Chemical Co, Chiba 299-0295, Japan

[5]Platform Laboratory for Science & Technology, Asahi KASEI Corporation, Shizuoka 416-8501, Japan

[6]Science & Innovation Center, Mitsubishi Chemical Corporation, Kanagawa 227-8502 Japan

[7]Research Center for Macromolecules and Biomaterials, National Institute for Materials Science, Ibaraki 305-0047, Japan

[8]Research Network and Facility Services Division, National Institute for Materials Science, Ibaraki 305-0044, Japan

[9]Research Center for Materials Nanoarchitectonics (MANA), National Institute for Materials Science, Ibaraki 305-0044, Japan

Table S1. Homo-PP samples.

sample ID	melt flow rate (g/10min)	weight-average molecular weight	molding temperature (℃)
1	0.55	919000	30
2	0.55	919000	45
3	0.55	919000	60
4	0.55	919000	70
5	0.55	919000	75
6	0.96	842000	30
7	0.96	842000	45
8	0.96	842000	60
9	0.96	842000	70
10	0.96	842000	75
11	2.46	576000	30
12	2.46	576000	45
13	2.46	576000	60
14	2.46	576000	70
15	2.46	576000	75
16	3.03	575000	30
17	3.03	575000	45
18	3.03	575000	60
19	3.03	575000	70
20	3.03	575000	75
21	4.66	465000	30
22	4.66	465000	45
23	4.66	465000	60
24	4.66	465000	70
25	4.66	465000	75
26	7.35	420000	30
27	7.35	420000	45
28	7.35	420000	60
29	7.35	420000	70
30	7.35	420000	75

31	8.49	422000	30
32	8.49	422000	45
33	8.49	422000	60
34	8.49	422000	70
35	8.49	422000	75
36	9.59	369000	30
37	9.59	369000	45
38	9.59	369000	60
39	9.59	369000	70
40	9.59	369000	75
41	9.38	415000	30
42	9.38	415000	45
43	9.38	415000	60
44	9.38	415000	70
45	9.38	415000	75
46	7.83	410000	30
47	7.83	410000	45
48	7.83	410000	60
49	7.83	410000	70
50	7.83	410000	75
51	8.33	394000	30
52	8.33	394000	45
53	8.33	394000	60
54	8.33	394000	70
55	8.33	394000	75
56	11.6	389000	30
57	11.6	389000	45
58	11.6	389000	60
59	11.6	389000	70
60	11.6	389000	75
61	13.4	371000	30
62	13.4	371000	45
63	13.4	371000	60
64	13.4	371000	70
65	13.4	371000	75
66	23.4	350000	30

67	23.4	350000	45
68	23.4	350000	60
69	23.4	350000	70
70	23.4	350000	75
71	32.1	287000	30
72	32.1	287000	45
73	32.1	287000	60
74	32.1	287000	70
75	32.1	287000	75
76	41.3	272000	30
77	41.3	272000	45
78	41.3	272000	60
79	41.3	272000	70
80	41.3	272000	75
81	102	200000	30
82	102	200000	45
83	102	200000	60
84	102	200000	70
85	102	200000	75

Table S2. Homo-PP with elastomer samples.

sample ID	melt flow rate (g/10min)	weight-average molecular weight	molding temperature (°C)	proportion of homo-PP (%)	screw type	rotational speed (rpm)
1	32.7	347000	45	90	S1	100
2	32.8	356000	45	90	S1	300
3	32.7	316000	45	80	S1	100
4	33.1	336000	45	80	S1	300
5	33.7	318000	45	70	S1	100
6	34	316000	45	70	S1	300
7	31.2	339000	45	90	S2	100
8	31.8	314000	45	90	S2	300
9	32.9	317000	45	80	S2	100
10	33.5	323000	45	80	S2	300
11	33.4	303000	45	70	S2	100
12	33.9	295000	45	70	S2	300
13	31.5	356000	45	90	S3	100
14	34.8	338000	45	90	S3	300
15	32.3	319000	45	80	S3	100
16	35.8	298000	45	80	S3	300
17	33.3	290000	45	70	S3	100
18	35.5	288000	45	70	S3	300
19	24.8	346000	45	90	S1	100
20	25.2	347000	45	90	S1	300
21	20.7	354000	45	80	S1	100
22	20.7	342000	45	80	S1	300
23	17.2	337000	45	70	S1	100
24	17.3	346000	45	70	S1	300
25	25.5	336000	45	90	S2	100
26	25.8	340000	45	90	S2	300
27	20.9	332000	45	80	S2	100
28	21.1	332000	45	80	S2	300
29	16.4	335000	45	70	S2	100
30	17.7	346000	45	70	S2	300

31	26.4	352000	45	90	S3	100
32	30.1	344000	45	90	S3	300
33	20.9	338000	45	80	S3	100
34	22.2	333000	45	80	S3	300
35	16.7	337000	45	70	S3	100
36	18.5	323000	45	70	S3	300
37	25.2	359000	45	90	S1	100
38	24.3	326000	45	90	S1	300
39	20.5	364000	45	80	S1	100
40	20.8	332000	45	80	S1	300
41	16.0	359000	45	70	S1	100
42	17.2	368000	45	70	S1	300
43	25.4	373000	45	90	S2	100
44	27.1	366000	45	90	S2	300
45	19.7	409000	45	80	S2	100
46	21.1	348000	45	80	S2	300
47	21.9	359000	45	70	S2	100
48	16.2	338000	45	70	S2	300
49	18.4	368000	45	90	S3	100
50	27.0	351000	45	90	S3	300
51	19.8	345000	45	80	S3	100
52	21.7	368000	45	80	S3	300
53	16.4	360000	45	70	S3	100
54	18.7	342000	45	70	S3	300
55	30.1	355000	45	90	S1	100
56	28.2	370000	45	90	S1	300
57	27.4	321000	45	80	S1	100
58	26.9	330000	45	80	S1	300
59	24.2	318000	45	70	S1	100
60	24.7	315000	45	70	S1	300
61	29.4	348000	45	90	S2	100
62	31.8	363000	45	90	S2	300
63	26.9	358000	45	80	S2	100
64	27.9	317000	45	80	S2	300
65	25.4	335000	45	70	S2	100
66	26.7	320000	45	70	S2	300

67	29.9	354000	45	90	S3	100
68	31.7	351000	45	90	S3	300
69	27	347000	45	80	S3	100
70	28.2	350000	45	80	S3	300
71	24.5	325000	45	70	S3	100
72	27.6	344000	45	70	S3	300
73	32.8	318000	45	90	S1	100
74	33.8	367000	45	90	S1	300
75	36.3	310000	45	80	S1	100
76	36.6	352000	45	80	S1	300
77	36.4	314000	45	70	S1	100
78	38.7	319000	45	70	S1	300
79	33.8	355000	45	90	S2	100
80	35.2	371000	45	90	S2	300
81	35.6	324000	45	80	S2	100
82	36.1	334000	45	80	S2	300
83	36.9	282000	45	70	S2	100
84	43.8	298000	45	70	S2	300
85	33.8	360000	45	90	S3	100
86	36.7	336000	45	90	S3	300
87	35.0	325000	45	80	S3	100
88	36.3	322000	45	80	S3	300
89	37.4	313000	45	70	S3	100
90	38.7	292000	45	70	S3	300
91	8.6	455000	45	80	S1	100
92	10.3	455000	45	80	S1	300
93	11.5	433000	45	80	S1	100
94	13.1	427000	45	80	S1	300
95	15.2	418000	45	80	S1	100
96	18	396000	45	80	S1	300
97	14.5	431000	45	80	S1	100
98	15.2	411000	45	80	S1	300
99	8.83	454000	45	80	S1	100
100	9.54	447000	45	80	S1	300
101	4.51	529000	45	80	S1	100
102	5.42	502000	45	80	S1	300

103	6.5	503000	45	80	S1	100
104	7.21	496000	45	80	S1	300
105	8.74	486000	45	80	S1	100
106	9.65	488000	45	80	S1	300
107	8.82	497000	45	80	S1	100
108	7.85	485000	45	80	S1	300
109	4.57	508000	45	80	S1	100
110	5.34	485000	45	80	S1	300
111	0.96	824000	45	80	S1	100
112	1.26	776000	45	80	S1	300
113	1.38	722000	45	80	S1	100
114	1.75	714000	45	80	S1	300
115	1.86	766000	45	80	S1	100
116	2.31	711000	45	80	S1	300
117	1.78	775000	45	80	S1	100
118	1.04	716000	45	80	S1	300
119	0.92	773000	45	80	S1	100
120	1.23	767000	45	80	S1	300

Table S3. R^2 values for the predictions using the 10-fold cross-validation when all the descriptors are extracted from the Bayesian spectral deconvolution technique for the homo-PP and the homo-PP-with-elastomer datasets.

	homo-PP	homo-PP-with-elastomer
tensile modulus	0.146	-0.641
yield stress	0.932	-0.229
yield strain	0.091	-5.087
elongation at break	-1.463	-40.917
flexural strength	0.550	-1.193
flexural modulus	0.238	-2.697
Charpy	0.724	-2.425
heat distortion	0.783	-3.614
Rockwell	0.425	-51.255

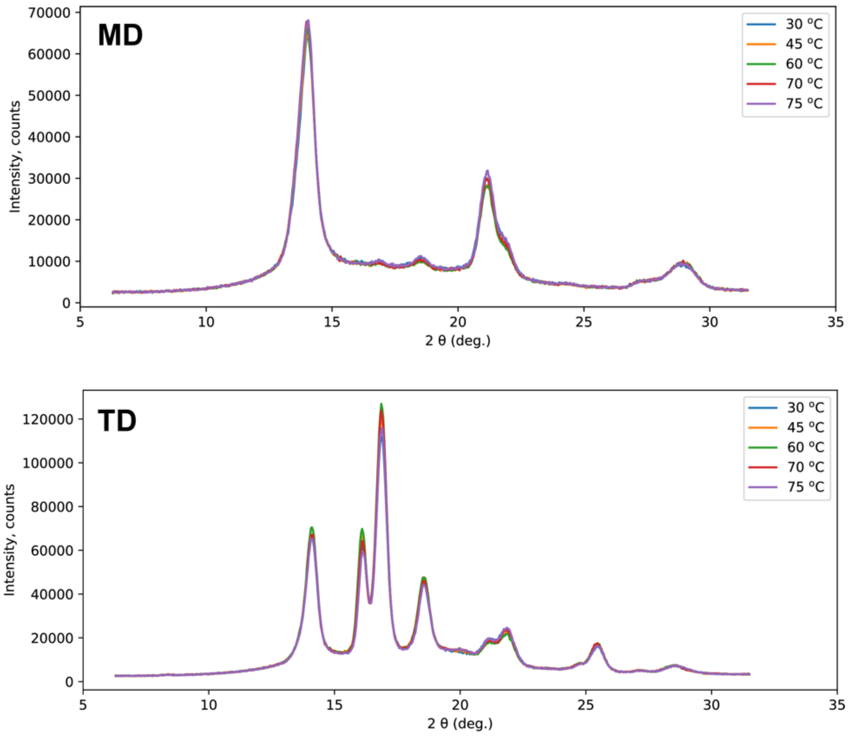

Fig. S1. Temperature dependence on XRD results in the injection molding process for homo-PP. The target samples are the ID 1 for each dataset.

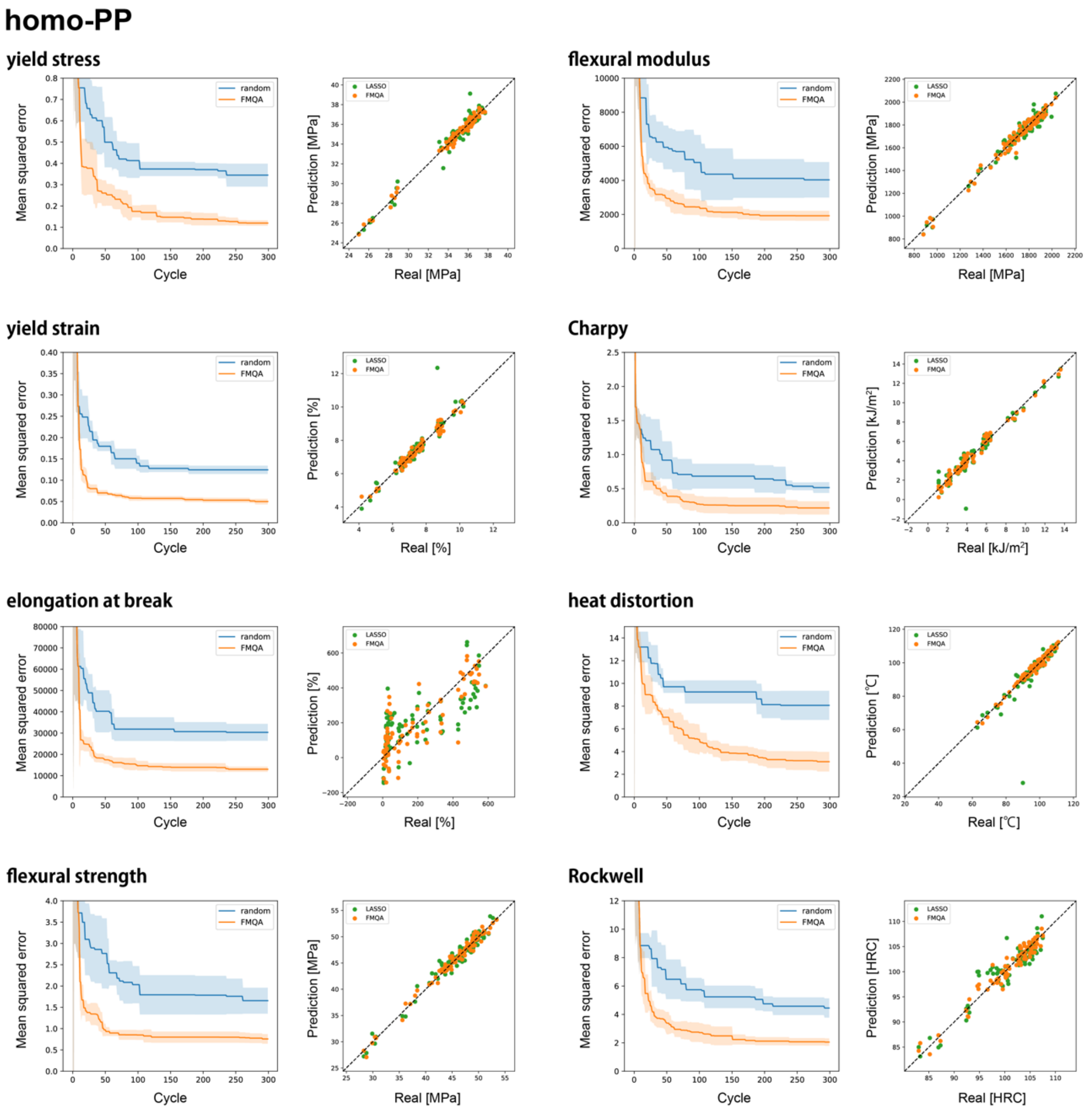

Fig. S2. Mean squared error (MSE) as a function of cycle number and prediction results for the homo-PP dataset. The MSE is evaluated via 10-fold cross-validation. The lines and shaded areas are the mean and standard deviation of five independent trials, respectively. For comparison, the LASSO results are also plotted.

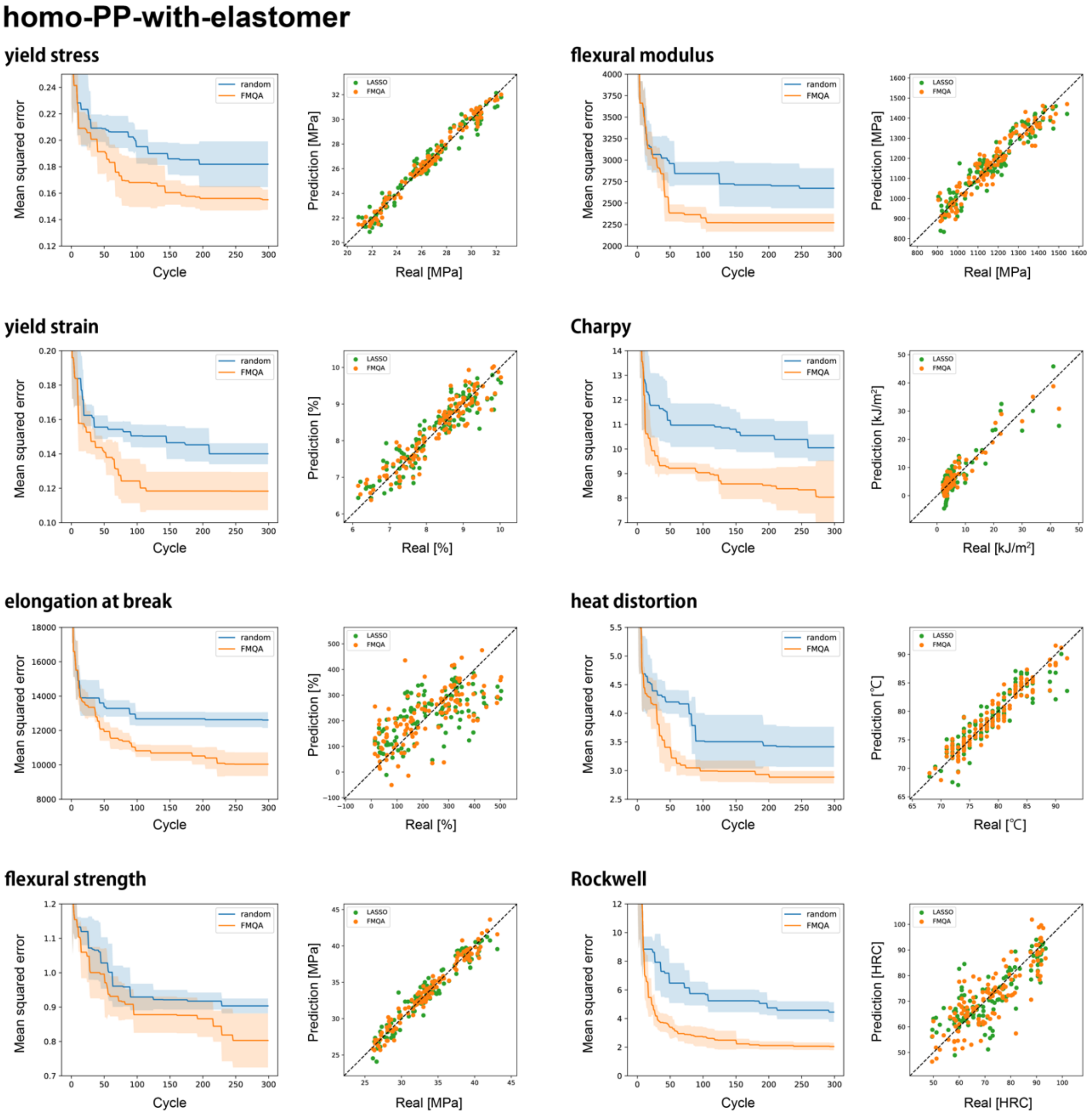

Fig. S3. MSE as a function of cycle number and prediction results for the homo-PP-with-elastomer dataset. The MSE is evaluated via 10-fold cross-validation. The lines and shaded areas are the mean and standard deviation of five independent trials, respectively. For comparison, the LASSO results are also plotted.

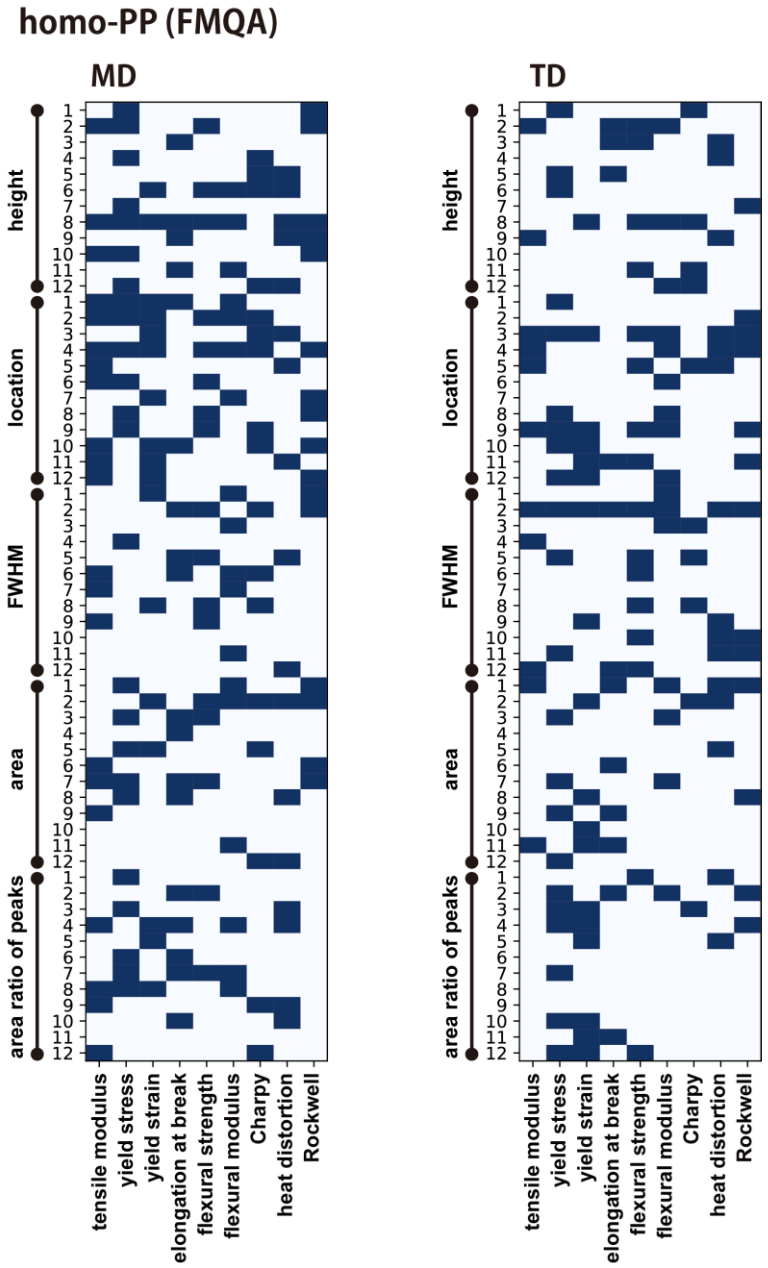

Fig. S4. Selected descriptors by FMQA for the homo-PP dataset depending on the mechanical properties. The blue points indicate the selected descriptors.

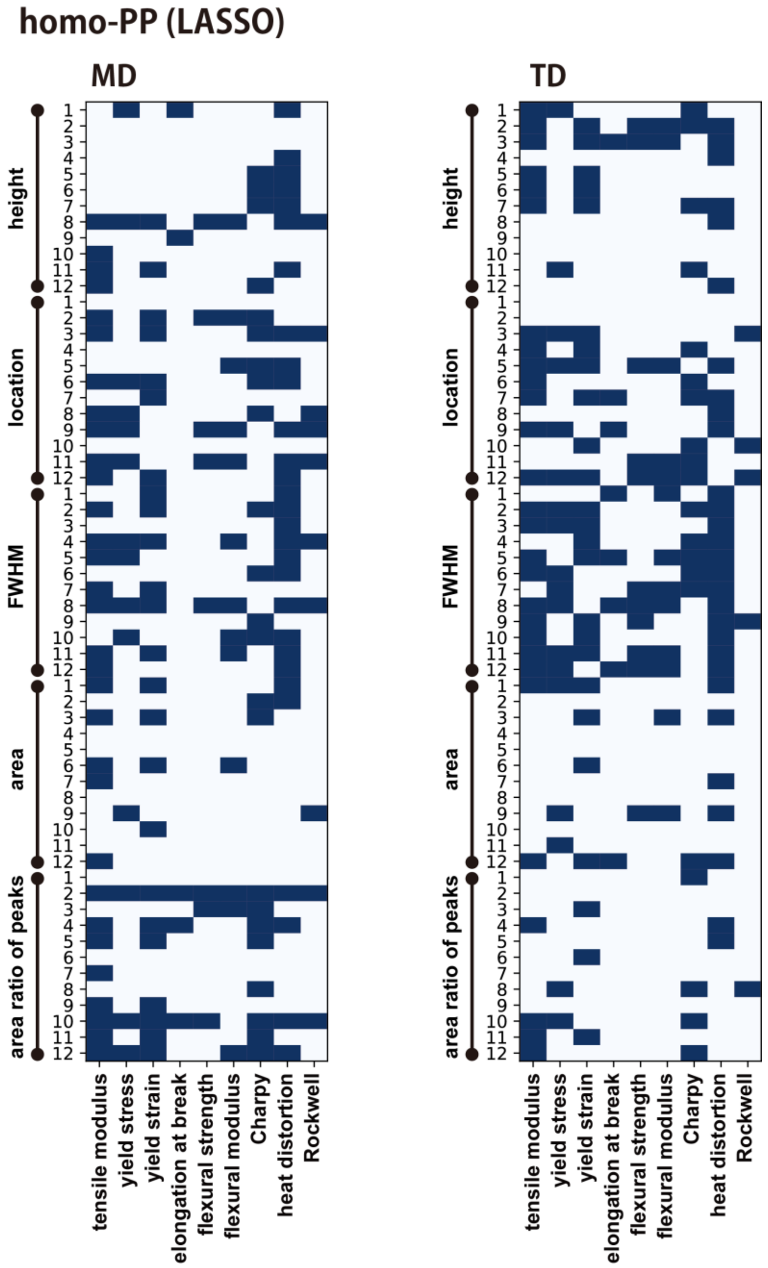

Fig. S5. Selected descriptors by LASSO for the homo-PP dataset depending on the mechanical properties. The blue points indicate the selected descriptors.

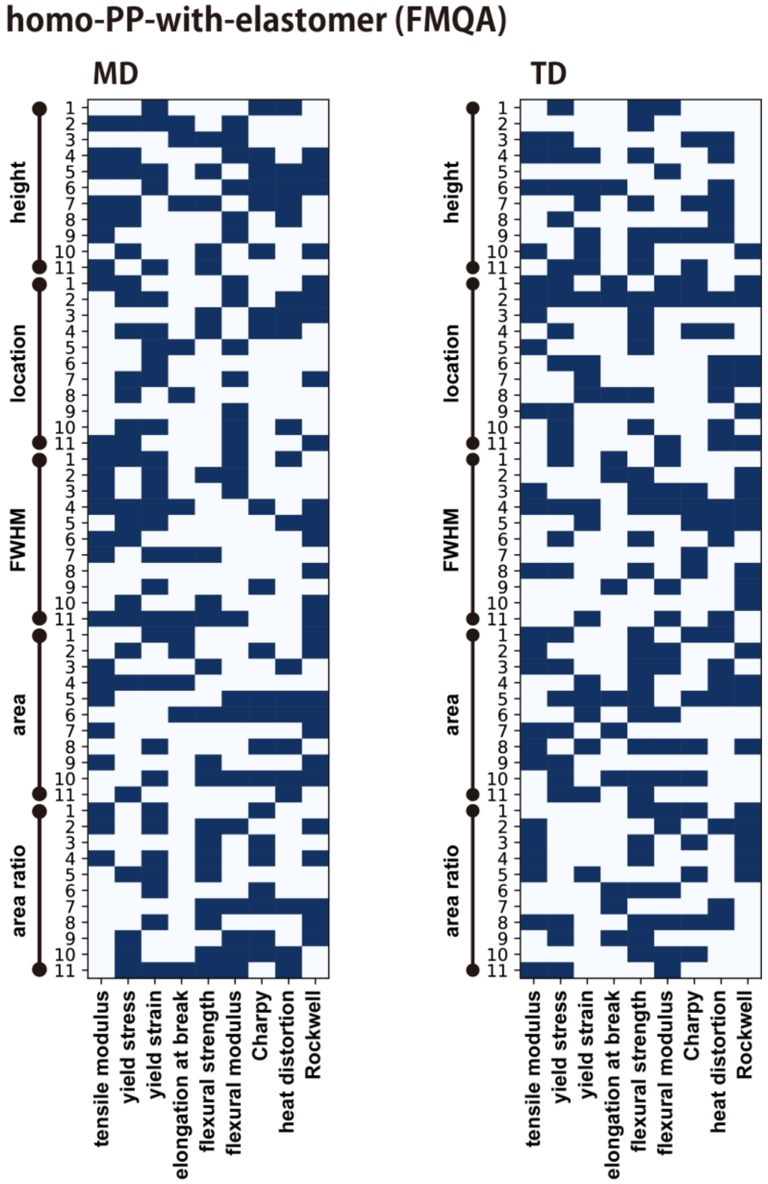

Fig. S6. Selected descriptors by FMQA for the homo-PP-with-elastomer dataset depending on the mechanical properties. The blue points indicate the selected descriptors.

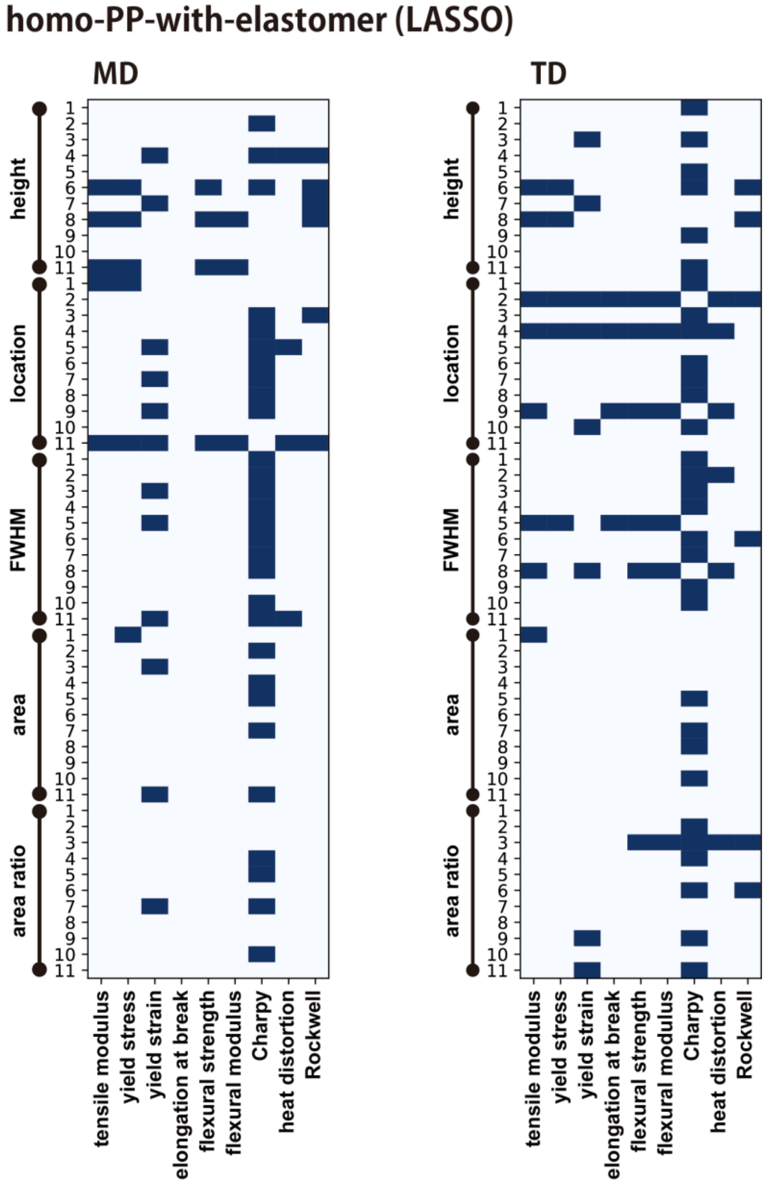

Fig. S7. Selected descriptors by LASSO for the homo-PP-with-elastomer dataset depending on the mechanical properties. The blue points indicate the selected descriptors.
